# Developing medical genetics in a low-income country: Unveiling the journey of the Pakistani Society of Medical Genetics and Genomics (PSMG)

**DOI:** 10.1016/j.gimo.2024.101883

**Published:** 2024-08-07

**Authors:** Aisha Furqan, Syed A. Ahmed, Rizwan Naeem, Myla Ashfaq

**Affiliations:** 1 Stanford Health Care, Palo Alto, CA; 2 Pakistani Society of Medical Genetics and Genomics, Manteca, CA; 3 Department of Genetics, Kaiser Permanente, Riverside, CA; 4 Department of Pathology, Albert Einstein College of Medicine, New York, NY; 5 Department of Pediatrics, Division of Medical Genetics, University of Texas Health Science Center at Houston (UTHealth Houston) and Children’s Memorial Hermann Hospital, Houston, TX

**Keywords:** Genetic counseling, Genetic testing, Pakistan, Pakistani Society of Medical Genetics, Telemedicine

## Introduction

As clinical and laboratory genetics services flourish in the Western Hemisphere, countries such as Pakistan are poised to step into this realm. However, despite the pressing need for advanced health care, the current scenario in Pakistan reveals a stark reality. With a population of approximately 220 million, the country allocates a mere 0.4% of its gross domestic product to health care, significantly below the World Health Organization recommended threshold of 6% for low-income countries.^1^^,^^2^ The health care system in Pakistan is organized into primary (ie, basic medical services, including immunizations, maternal care, and treatment of common illnesses), secondary (ie, specialized, including surgery, diagnostic services, and specialist consultations), and tertiary care centers (advanced medical services, specialized treatments, major surgeries, etc) that are run by nongovernment organizations (NGOs), government, and private entities.^3^ The financial coverage of health care varies widely between the different organizations from no-cost provision by government and NGO run facilities to high out-of-pocket cost for privately run facilities. According to data from the Pakistan Demographic and Health Survey conducted in 2017 and 2018, only about 1% of women and 4% of men in Pakistan have any form of health insurance.^4^ Additionally, although government-sponsored health insurance schemes, such as the Prime Minister’s National Health Program and the Health Insurance Scheme for the Poor, aim to expand health care coverage to low-income populations, specific coverage for genetic services are not explicitly outlined in these programs largely because of the lack of availability of such services at the population level (eg, population-wide prenatal or new-born screening programs).^5^

Although medical genetic services are not as widely integrated into the health care system, larger cities have established some medical genetics services at academic institutions, including Aga Khan University (AKU) in Karachi, Shifa College of Medicine, Pakistan Institute of Medical Sciences in Islamabad, and Gomal University in Dera Ismail Khan. Although specific departments dedicated solely to medical genetics may not exist, departments such as pathology or pediatrics incorporate sections dedicated to molecular pathology and medical genetics at King Edward Medical University and Allama Iqbal Medical College in Lahore. Additionally, the government of Pakistan, has initiated public health programs targeting certain genetic disorders that offer cascade screenings for relatives of individuals with Beta Thalassemia (eg, Punjab Thalassemia and Other Genetic Disorders Prevention and Research Institute).^6^ Of note, the College of Physicians and Surgeons Pakistan, a professional body responsible for the certification and postgraduate medical training, does not currently offer a medical genetics specialty, and the Higher Education Commission, the entity that accredits postgraduate-level degrees, does not have an accredited genetic counseling training program in the country. As a result, the lack of medical genetics experts continues to hinder access to genetic services at the population level.^6^ In this context, genetic testing is often perceived as a luxury, not a necessity. However, with high levels of consanguinity, infant mortality, and genetic disorders prevalent in the country, medical genetics services are becoming increasingly crucial.^6^^,^^7^ Establishment of infrastructure for education, genetic testing, and clinical genetics facilities is necessary to improve healthcare outcomes in Pakistan.

In recognition of these challenges, the Pakistani Society of Medical Genetics and Genomics (PSMG) emerged in the summer of 2020, initiated by 2 genetic counselors and 2 geneticists of Pakistani origin residing in the United States. The founding members personally experienced the lack of medical genetics training in medical schools and graduate universities in Pakistan. They recognized the importance of establishing genetics clinics and providing training to health care providers to address this issue. Additionally, they witnessed the struggles of individuals living with genetic disorders who had difficulty accessing medical genetic services for diagnosis and management of their conditions. As individuals who received their early professional education in Pakistan, they felt a sense of responsibility to give back to their home country.

In the early days of the COVID-19 pandemic, international travel was restricted because shelter-in-place advisories. As a result, people became more familiar with virtual meetings, which proved to be helpful for collaborative efforts in establishing the Society. The team experimented with different models of application and decided on weekly Zoom meetings with core founding members, inviting thought leaders and stakeholders for discussion and guidance, inviting volunteer speakers for online education, initiating telehealth genetics clinics in pediatric and prenatal settings, and recruiting an undergraduate student to manage the social media platform for outreach.

Based on the previously documented needs assessment, PSMG formulated specific goals to (1) increase the genetics workforce through education and training, (2) enhance clinical genetics services and increase access to genetic testing, and (3) increase awareness of medical genetics and provide support for individuals with genetic disorders.^6^

This commentary aims to chronicle the organization’s goals, achievements, and future directions, providing valuable insights into the progress made thus far and offering a model framework for other countries facing health care challenges similar to Pakistan.

### Educational efforts

Medical genetics education in Pakistan is currently at a rudimentary stage, lacking postgraduate degrees in clinical genetics, laboratory genetics, or genetic counseling.^1^^,^^8^ Furthermore, genetics education in medical school requires further bolstering because Pakistani medical students report desiring additional genetics education in the areas of genetic testing, cancer genetics, and treatment of genetic disorders to aid in their role as future physicians.^1^

To address the shortage of health care professionals with genetics expertise, several options are being explored, including offering specialty-specific certificates, 6-month certificate programs, and short courses for medical doctors and nurses. Although these are plausible options, the Society decided to focus its efforts on establishing a 2-year Master’s in Genetic Counseling program similar to those in North America and other parts of the world. To gain insights into the process of setting up a new program, we met with key stakeholders, who were instrumental in similar endeavors in other countries. This collaborative approach enabled us to appreciate both the challenges and successes faced by other groups, propelling us to share our own experiences.

Although no official market studies have been conducted on the subject, the organization has received significant interest in establishing the program via needs assessment survey conducted in 2022 (ie, 91% of surveyors responded, “yes” to “Should formal genetic counseling training be available in Pakistan?”) and continued feedback during the Q&A sessions of the online lecture series.^6^ In addition, several inquiries have been received via email from interested parties in Pakistan, demonstrating a strong interest in the program. Furthermore, one of the PSMG board members, who is a PhD geneticist in Lahore, mentioned that several PhD genetics students at their university would be highly interested in enrolling in the program if it is initiated. On the note of employment, the 2 individuals that received on-the-job training are in high demand for their unique knowledge and skill set. As more genetic testing becomes available and affordable in the country, the need for genetic counselors will increase to better understand the results and their implications on the affected families.

As an initial step for program development, PSMG reached out to representatives from various public and private postgraduate educational institutions to identify a local partner institution that could host the program. Several institutions across different cities expressed interest. To kickstart this process, a tentative 2-year genetic counseling curriculum was designed with respect to cultural and societal norms along with the medical genetics didactics by combining the GC curricula from Western and Middle Eastern GC programs. For instance, the 4-semester long psychosocial course work would aim to address the cultural and religious attitudes and beliefs toward genetic disorders. In partnership with local institutions, some courses will be taught by local educators who specialize in molecular genetics, biomedical ethics, public health, and religious aspects of genetic counseling. Other courses that focus on the medical training and psychosocial counseling aspects will be taught by US-trained genetics experts via virtual synchronous or asynchronous lectures. To assist in faculty identification, members of the organization-initiated efforts to compile a list of practicing genetic counselors in the United States with Pakistani origins who may be interested in serving as faculty members or contributing to various roles in a future genetic counseling program in Pakistan. Presently, we have identified 23 individuals in the United States who are either practicing genetic counselors or are currently enrolled in a genetic counseling program. These individuals are members of the Society and maintain communication through a WhatsApp group.

Other considerations that need to be addressed are establishing clinical rotation sites in the absence of genetics clinics and identifying faculty capable of teaching genetic counseling course content. To address the issue of not having many local clinical rotation sites, PSMG established clinical relationships with medical institutes in Pakistan (further described below). In this arrangement, members of the organization volunteered their genetic counseling expertise in concert with local medical teams via telehealth, with the expectation that these clinics would serve as training sites for future genetic counseling trainees.

Stakeholders from multiple higher education institutions in Karachi, Lahore, Islamabad, and Dera Ismail Khan were invited to discuss potential collaboration on educational efforts. However, the collaboration faced several barriers, including lack of follow-through from educational institutions that would house the postgraduate training programs. The most cited reason for this lack of follow-through was shortage of funding and recruitment for students for training in genetics when no employment opportunities existed, as well as administrative time allotment to complete the paperwork. Some programs asked for large class sizes to gather larger sums of tuition to sustain the program in the initial stages. However, this request was tabled to maintain adequate standards of training in limited clinical rotation opportunities available in the country. Despite these challenges, we continue to work on establishing partnerships between higher education institutions, including AKU, Pakistan Institute of Medical Sciences, and others.

Although these efforts are ongoing, in the interim, PSMG hosted virtual educational seminars on medical genetics topics for health care professionals and the public to strengthen genetics knowledge and awareness. These were introductory-level lectures provided by genetics experts from the United States and Pakistan. US-based PSMG members and volunteers provided their expertise for the educational activities, whereas local collaborators in Pakistan helped to market and spread the word about the activities. Some stakeholders also provided physical locations, including conference rooms, for students and placed adverts on their websites with login information. Support group organizations, such as Special Needs Pakistan and Karachi Down Syndrome Program, posted adverts of the educational activities, and parents of the affected individuals took part in Q&A sessions. This lecture series has had robust attendance and continues to serve as a means to fill gaps for Pakistani health care providers regarding genetic testing and genetic counseling. The question-and-answer session at the end of these discussions provides further evidence regarding the interest and desire of the participants to learn more about genetics. In addition, videos of these lectures are available for viewing on YouTube and can be easily accessed through PSMG website (www.psmg.org).

Additionally, inspired by an example of an on-the-job training model for genetic counseling training by a local medical geneticist, members of PSMG trained 2 clinic coordinators through virtual classes and observations of telehealth genetics clinics. Although this effort is on an extremely small scale, these 2 providers are now able to carry out basic genetic counseling sessions, including pre- and posttest counseling, order genetic testing under physician guidance, and serve as genetics experts who can address medical genetics queries at their respective institutions. Additionally, these individuals can assist in the training of future local genetic counselors.

The authors of the article continue to make periodic trips to Pakistan to have in-person discussions with local health care providers collaborating with PSMG and gave guest lectures to practicing and trainee physicians on various topics including medical genetics and genetic counseling. During their visits, they were provided with tours of the facilities at National Institute of Child Health where telehealth genetics clinics take place and testing services are provided. More recently, PSMG collaborated with the AKU Hospital to organize a hybrid educational symposium titled “Practical Guide to Medical Genetics: Incorporating Genetics into Everyday Medical Practice,” held in Karachi on February 3rd, 2024. This hybrid event featured presentations from both local and international physicians and genetic counselors. The symposium stimulated engaging discussions on various topics, including health literacy, perspectives on consanguinity, and the impact of cultural, religious, and societal beliefs on medical practices. The discussions underscored the importance of surveying health care professionals in Pakistan to gather feedback on effective communication strategies with patients, considering the aforementioned factors.

Lessons learned:

1. A professional organization can help stir interest and awareness of the importance of medical genetics. Other countries could follow suit and establish their own professional society as a concrete way of advocating for the integration of medical genetics in their own setting.
2. Although establishing a Master’s degree in Genetic Counseling is a long and arduous task, other educational initiatives can be taken in the interim to disperse knowledge and create awareness. For instance, PSMG organized a series of webinars that are available for viewing on the PSMG YouTube channel and continue to provide on-the-job training to the clinic coordinators in genetic counseling techniques.
3. Genetic counseling training in countries such as Pakistan will diverge from its Western counterparts due to various factors. The approach will heavily rely on local resources, encompassing the available institutional infrastructure, governmental regulations, existing medical facilities, and cultural norms prevalent in the country. The key lies in adapting and tailoring existing models to suit the specific needs and contexts of Pakistan. By leveraging the resources already in place and customizing them to align with local requirements, the training can be effectively tailored to meet the unique demands of the country.
4. The Society appreciates that the creation of culturally appropriate educational content will be an ongoing process as we continue to understand societal and individual values used in medical decision making by the Pakistani patient population.

### Clinical efforts

At the time of the inception of the Society in the summer of 2020, 2 foreign-trained geneticists were practicing in the urban city of Karachi as the sole genetics’ providers in Pakistan, suggesting a ratio of 1 geneticist to 7.5 million people.^6^ Although health care providers offer counseling in their specialties, such as obstetrics, pediatrics, cardiology, and oncology, there are no genetic counselors in Pakistan at this time with a Master’s degree in Genetic Counseling. As per the needs assessment webinar, health care providers in Pakistan emphasized the challenges of providing counseling to their patients with minimal training on the theoretical principles of medical genetics and virtually no training on counseling techniques.^6^ To the best of our knowledge, by the time of writing this manuscript, clinical genetics services are provided in some capacity in clinic settings in the cities of Karachi, Lahore, Islamabad, and Dera Ismail Khan, whereas no telehealth facilities are available. This gap in medical genetics service delivery has been reported by others and has been noted anecdotally by the authors.^9^

As a result, the founding members of the Society collaborated with neurologists, endocrinologists, gastroenterologists, and perinatologists to establish 2 telehealth genetics clinics for underserved populations of Pakistan. The originating sites are physically located in Karachi where patients and their families get treated for the genetic conditions. The telehealth genetics clinics embedded within the existing infrastructure of these tertiary care centers clinics are supported by (1) attending physicians, who present the case summary, (2) clinic coordinators, who collect the medical and family histories of the patients, and (3) the US-based medical genetics team, who provide consultation to the local team. A unique Zoom link is shared with the involved parties to avoid any unintended privacy concerns. Patients and their families are provided with options to either join the Zoom call from the originating sites or their remote locations (home, office, etc). The health care providers from the originating sites brief the US-based team on the medical and family history of the proband and review the case ahead of the scheduled appointment via Zoom. During the appointment, providers from the originating sites engage with the patient, whereas the US-based genetics experts provide counseling and anticipatory guidance. After the visit, the providers from the originating sites complete documentation, arrange follow-up visits for cascade testing, and suggest referrals to other specialties when required. Local providers also seek advice on complex genetics cases and interpretation of genetic testing results for patients who are unable to attend telehealth clinics because of scheduling conflicts. It is important to note that US-based genetics experts provide their expertise at no cost, during their nonworking hours, and their US employers do not cover their time or provide indemnity. In the 2 years since the start of the biweekly telehealth clinics, the team has seen a wide range of cases from challenging diagnostic odysseys to complex psychosocial situations (manuscript is being prepared to publish the data).

Around 61% of Pakistan’s population lives in rural areas, where they have limited access to hospitals and tertiary care centers compared with their urban counterparts. In response to the COVID-19 pandemic, telemedicine was introduced into the country and has since been widely used through various smartphone applications in several parts of the country.^9^ Apart from the obvious economic benefits of telemedicine, the authors noted that patients who attended the sessions from their homes seemed more engaged and comfortable asking questions compared with those who traveled to the originating sites typically accompanied by relatives. Although in-depth post-session satisfaction surveys were not conducted, it is reasonable to assume that this ease of communication increases a sense of privacy, particularly when asking more private questions about reproduction and child-bearing. Patients may feel shy or embarrassed asking such questions in front of providers of the opposite gender or relatives who accompany them on their visits.

Furthermore, with a high-rate of first-degree consanguinity, the Pakistani population is a rich source for various kinds of genetic disorders necessitating compilation of population-specific databases. From the clinical experience through the telehealth genetics clinics, genetic testing revealed variants of uncertain significance as previously reported.^10^, ^11^, ^12^ In 2017, Qasim et al^13^ created a Pakistan Genetic Mutation Database to facilitate the gathering of population-specific variants. By submitting the variant curation data ascertained by telehealth clinics and other subspecialty clinics, Pakistan Genetic Mutation Database can be enriched with valuable data for variant interpretation.^13^

Based on these observations, future research could explore the possibility of increased uptake of genetic testing if the results could be kept private and not shared with relatives who might harm the social or physical well-being of the tested person. In summary, this highlights the need for alternative delivery models, particularly concerning privacy, while still maintaining the shared decision-making dynamics of Pakistani families.

Lessons learned:

1. Health care providers, medical students, and genetic counseling trainees are interested in learning genetic counseling techniques to explain abstract concepts to patients. Because of limited health literacy and language limitations, such as a lack of literal translations of words such as “genes” and “chromosomes” in Urdu, the use of analogies is helpful during consultations. One of the initiatives by the Society is to create visual aids to help with genetic counseling in Urdu and other local languages.
2. As part of informed consent in pretest settings, ethical issues concerning testing unaffected minors, shame/guilt, termination of affected pregnancies, etc. are appreciated by the genetic counseling trainees. Although certain psychosocial issues encountered during the telehealth visits remained similar between the United States and Pakistani populations, many psychosocial issues were unique to the Pakistani population particularly around decisions of cascade testing and sharing of information among at-risk relatives and lack of implementation of privacy protection laws.
3. Despite initial challenges with IT difficulties, the telehealth genetics clinics have been successful as a delivery model to reach patients in remote parts of the country and have also facilitated in maintaining privacy concerns of many patients. Future researchers could explore the utility of telehealth as a delivery model for medical genetics consults in detail.
4. A population-specific variant database with clinical variant curation can facilitate better understanding of unique variants not previously seen in other populations. Thus, aiding in the broader knowledge base of the scientific community and reducing the rate of variants of uncertain significance encountered in clinical settings.

### Laboratory relationships

Cost remains a significant hurdle to widespread genetic testing adoption within the Pakistani population. A substantial portion of genetic tests are currently outsourced, compounding expenses due to shipping samples abroad and prolonging turnaround times. For instance, a European commercial lab is advertising exome sequencing at $299 (approximately PKR 83,687.52 based on current conversation rates). To put this in context, the average annual household income per capita in Pakistan was $587.069 in June 2019.^14^ This shows that an average family would have to spend more than half of their annual income to obtain the test.

Notably, a prominent cancer hospital in the country is addressing cost challenges by offering hotspot testing for pathogenic variants in *BRCA1* and *BRCA2* genes prevalent in the population, aiming to enhance diagnostic yield at a more affordable rate.^15^ Additionally, academic institutes in Pakistan collaborate with US and European laboratories for individuals suspected of having inborn errors of metabolism, showcasing a high diagnostic yield for exome sequencing testing.^12^

PSMG facilitated connections between clinicians in telehealth genetics clinics and US-based labs, conducting numerous tests with a particular focus on providing no-cost testing to at-risk relatives. This initiative proved invaluable for many families, as indicated by unpublished data. To mitigate expenses, the originating sites’ staff reduced sample shipment costs by consolidating multiple samples in the same container. Ongoing efforts involve seeking global partnerships to ensure accessible and high-quality genetic testing for the Pakistani population.

Lessons learned:

1. Reducing the cost barrier significantly enhances genetic testing uptake among Pakistanis. For instance, payment installments for large bills are available at a local lab that currently outsources molecular genetic testing to European labs.
2. Although obtaining a Master’s degree in Genetic Counseling remains the ultimate objective, providing training to essential medical professionals on the mechanics of ordering genetic testing can yield significant benefits. This training would encompass understanding the utility of genetic testing, the different types of tests available, and the process of pre- and posttest counseling. Clinic coordinators trained by the PSMG team have successfully assumed this responsibility, ensuring the proper administration of genetic testing procedures.
3. The challenge of interpreting genetic test results, particularly in non-White populations with numerous variants of uncertain significance, necessitates educational workshops and seminars for clinicians. These sessions focus on variant interpretation and curation processes to empower ordering providers in navigating and concluding genetic diagnostic journeys for their patients. The PSMG team continues to provide variant interpretation workshops to interested clinicians in those settings where PSMG does not provide telehealth clinical expertise (eg, at the National Institute of Cardiovascular Disease in Karachi).

### Community outreach

Within the last decade, driven by increased access to affordable smartphones and improved telecommunications infrastructure, 165 million Pakistanis are connected to the internet. Taking advantage of this, having a presence on social media is an ideal and efficient way to reach the masses.^16^ PSMG has successfully established its online presence through the development of a dynamic website featuring the latest updates, publications, and educational videos. Within the last 12 months (May 2023-May 2024), the PSMG website had 3472 visitors and 210 visitors in the last 30 days (April 2024). The PSMG YouTube channel had 260 views during the last 365 days (May 2023-May 2024). Complementing this, an Instagram page was launched to foster connections with like-minded organizations and enhance visibility. As of writing this commentary, we had 155 followers on Instagram. Furthermore, we have actively engaged with patient support organizations, including Special Needs Pakistan and the Karachi Down Syndrome Program. Members from these organizations actively participated in our webinars, and there are ongoing plans for collaborative research projects between PSMG and these valued patient support organizations.

Lessons learned:

1. Having an online presence is a necessary initial step for the creation of an organization as this enables reaching a broader audience, establishing credibility, and facilitating communication and interaction with potential stakeholders or members.
2. Establishing relationships with patient advocacy and support organizations provides invaluable insights into the needs and views of patient populations, as well as enhancing the impact of initiatives aimed at addressing these needs.

### Future directions

Although the organization has made significant strides in the 3 and a half years since its inception, there is still much that needs to be achieved. In terms of education, at the time of the writing of this commentary, efforts are underway to form partnerships with local educational institutions to establish a Master’s degree in Genetic Counseling. In the interim, we will continue to promote genetics education through online lecture series, case conferences, genetics conferences, symposiums, etc. In the clinical realm, in addition to providing consultation services to local physicians, we will aim to promote access to genetic services to the masses by using telehealth as an alternate delivery model. For laboratory services, we hope to eventually garner funding via donations as a 501(c)(3) organization, to be able to support patients who require genetic testing. Lastly, in terms of community outreach, our goal is to develop research projects with patient support organizations using their membership to ascertain the needs and views of parents of children with genetic disorders in Pakistan. We will continue to report and document our journey so that it can serve as a model for others looking for inspiration and guidance. Lastly, the PSMG volunteer members are starting to organize events and projects to bring awareness of genetic disorders in the country. These initiatives include organizing health fairs, online educational activities, writing articles for the Society’s newsletter, and translating patient education materials into Urdu and regional languages.

In conclusion, as a low-income country, genetic services remain unaffordable for the masses without major subsidies from the government and NGOs. PSMG can help alleviate some of the financial barriers by collaborating with the local government and NGOs and by raising funds to subsidize genetic testing for low-income families in need. This will help reduce the financial burden and ensure that essential genetic services are accessible to everyone, regardless of their economic status. It is important to note that the development of clinical genetic services in Pakistan will be a gradual process, similar to what has been observed in Western countries. The training of genetics professionals to serve the nation adequately will require time and investment. However, as local capacity increases and genetic testing becomes more readily available, there will likely be an increase in awareness and uptake of these services.

### Information regarding PSMG

PSMG is a 501(c)(3) organization registered in the state of California. According to the Internal Revenue System’s website, “Organizations described in section 501(c)(3) are commonly referred to as charitable organizations. Organizations described in section 501(c)(3), other than testing for public safety organizations, are eligible to receive tax-deductible contributions in accordance with Code section 170”^17^ The founding members of PSMG donate their time and resources to the organization. Our general membership is currently free of cost and consists of 133 members. The board of directors consists of 7 members (experts from Pakistan and the United States) and meets quarterly to discuss future goals and accomplishments. Detailed bylaws of the Society can be found on the website (www.PSMG.org).

## Conflict of Interest

Ms Furqan holds stocks in Natera, Inc, Aisha Furqan, Myla Ashfaq, Syed Ajaz Ahmed, and Rizwan Naeem are cofounders of the nonprofit Pakistani Society of Medical Genetics and Genomics. All the work done by the cofounders is on a voluntary bases, and they do not receive any monetary compensation.
